# *EINCR1* is an EGF inducible lincRNA overexpressed in lung adenocarcinomas

**DOI:** 10.1371/journal.pone.0181902

**Published:** 2017-07-21

**Authors:** Karol Nowicki-Osuch, Yaoyong Li, Mairi Challinor, David T. Gerrard, Neil A. Hanley, Andrew D. Sharrocks

**Affiliations:** 1 School of Biological Sciences, Faculty of Biology, Medicine and Health, University of Manchester, Manchester Academic Health Science Centre, Manchester, United Kingdom; 2 School of Medical Sciences, Faculty of Biology, Medicine and Health, University of Manchester, Manchester Academic Health Science Centre, Manchester, United Kingdom; 3 Endocrinology Department, Central Manchester University Hospitals NHS Foundation Trust, Manchester, United Kingdom; Oxford Brookes University, UNITED KINGDOM

## Abstract

Long non-coding RNAs are being increasingly recognised as important molecules involved in regulating a diverse array of biological functions. For example, many long non-coding RNAs have been associated with tumourigenesis and in this context their molecular functions often involves impacting on chromatin and transcriptional control processes. One important cellular control system that is often deregulated in cancer cells is the ERK MAP kinase pathway. Here we have investigated whether ERK pathway signaling in response to EGF stimulation, leads to changes in the production of long non-coding RNAs. We identify several different classes of EGF pathway-regulated lncRNAs. We focus on one of the inducible lincRNAs, EGF inducible long intergenic non-coding RNA 1 (*EINCR1*). *EINCR1* is predominantly nuclear and shows delayed activation kinetics compared to other immediate-early EGF-inducible genes. In humans it is expressed in a tissue-specific manner and is mainly confined to the heart but it exhibits little evolutionary conservation. Importantly, in several cancers *EINCR1* shows elevated expression levels which correlate with poor survival in lung adenocarcinoma patients. In the context of lung adenocarcinomas, *EINCR1* expression is anti-correlated with the expression of several protein coding EGF-regulated genes. A potential functional connection is demonstrated as *EINCR1* overexpression is shown to reduce the expression of EGF-regulated protein coding genes including *FOS* and *FOSB*.

## Introduction

Over recent years, long noncoding RNAs (lncRNAs) have emerged as a large and diverse group of genes. The discovery of these genes arose due to the advent of the next generation sequencing technologies which indicated that up to 80% of human genome is transcribed [1]. LncRNAs are defined as transcripts that are over 200 nucleotides long and do not contain protein coding open reading frames [2]. Functional studies have implicated lncRNAs in many cellular processes including the regulation of transcription, RNA processing, siRNA attenuation and RNA stabilisation [3]–[6]. Over recent years, lncRNAs have become prominent elements of regulatory networks associated with cell growth, differentiation and cancer progression. Many lncRNAs have been associated with controlling gene expression (reviewed in [7]) with arguably the best studied lncRNA being *MALAT1* and its role in cancer. For example, *MALAT1* was recently shown to be a critical factor in breast cancer metastasis [8]. These findings have triggered other studies into the role of lncRNAs in cancer. For example RNA-seq experiments in prostate cancer cells identified 121 novel lncRNA associated with that disease [9]. Further investigation of the most upregulated lncRNA, *PCAT1*, indicated that it is predominantly repressive in nature and it influences expression of genes associated with mitosis and cell cycle. Recently, it has been shown that transcription of *PCAT1* is regulated by a specific distal regulatory element (via ONECUT2 transcription factor) and that *PCAT1* itself is regulates expression of androgen receptor late response genes in LSD1-dependent manner [10]. The identification of additional lncRNAs whose expression changes in disease states or in response to cellular signalling would therefore likely identify new potential regulatory molecules that are important in signal-dependent processes.

The EGF signaling pathway and its effects on protein coding gene expression has been extensively studied and represents an excellent model system in which to identify and investigate the function of lncRNAs. For example previous studies using the human MCF10A breast epithelial cell line have identified dynamic changes in the expression of protein coding genes and miRNAs following EGF induction [11]. Protein coding genes which are upregulated are typically from the immediate-early class of genes including well studied genes like *FOS* and *EGR1*. Many of the genes activated by EGF signaling encode transcription factors that propagate downstream transcriptional events. However, a number of feedback loops are triggered that act to turn off or moderate the levels of the initial transcriptional wave, including a role for miRNAs [12]. The immediate-early genes are also activated by different growth factors and other mitogenic signals through activation of the ERK MAP kinase pathway [13]. Importantly, EGF receptor tyrosine kinases and the downstream ERK pathway are commonly upregulated in cancer [14], meaning that the identification of novel downstream effector molecules is potentially important for enhancing our understanding of the tumourigenic process. Here we used RNA-seq to identify EGF-regulated lncRNAs and identify several different classes of lncRNA. We focus on one of the lincRNAs, EGF inducible long intergenic non-coding RNA 1 (*EINCR1*), and characterise its transcriptional activation mechanism, its subcellular localisation, its expression profile and potential role in controlling gene expression.

## Results

### Identification EGF-inducible lncRNAs

**Fig 1:**
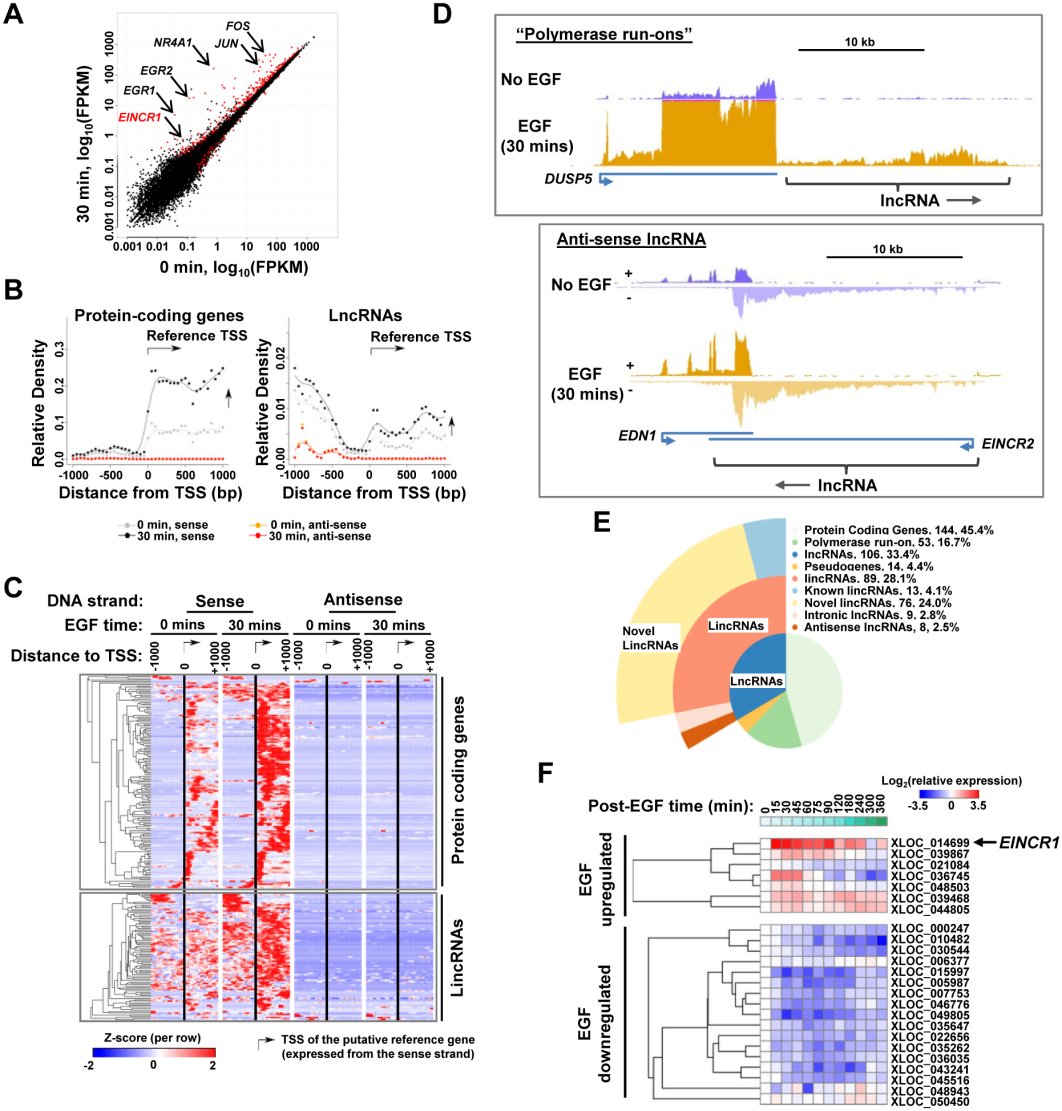
EGF-stimulation leads to transcriptional induction of lncRNAs. (A) Scatter plot of differential gene expression before (0 min) and after (30 min) EGF stimulation. The identities of several upregulated protein coding genes and the lincRNA *EINCR1* are indicated. (B) Average profiles of sequencing read density in the 2000 bp window around the putative transcription start sites (TSS) of EGF-upregulated protein-coding genes and lincRNAs. (C) Heat map showing the sequencing read density in the 2000 bp window around the putative transcription start sites (indicated by arrows) of EGF-upregulated protein-coding genes and lincRNAs. Data are row Z-normalised. (D) Example genome browser views of RNAseq reads located around the *DUSP5* (top) and *EDN1* (bottom) loci. LncRNAs located immediately downstream from *DUSP5* (“polymerase run on”) and on the anti-sense strand downstream from *EDN1* (*EINCR2*) are shown. (E) Distribution of EGF-upregulated genes across gene classes defined by the ENCODE project. The percentage of transcripts falling into each class is indicated. (F) RT-qPCR analysis of the expression of the indicated up- and down-regulated lincRNAs following EGF induction for the indicated times. Data are shown according to the indicated colour coding (capped at log_2_≤3.5) and are the average of three biological replicates (n = 3).

To identify lncRNAs that are inducible by EGF pathway signalling, we treated non-tumourigenic human breast epithelial MCF10A cells with EGF for 30 mins and performed RNA-seq. We focussed on the nuclear RNA fraction as many lncRNAs are known to be preferentially localised to this subcellular compartment [1]. We identified a total of 482 transcripts (>200 bp) that are upregulated or downregulated by EGF (log_2_ fold change >0.58, P-value <0.05), the majority of which are upregulated (Fig 1A; S1 Table). Well characterised protein coding genes such as *FOS* and *EGR1* are among the upregulated genes although lncRNAs are also upregulated including one which we have named *EINCR1*.

Next, we plotted the average sequencing reads found in a 2 kb region spanning the putative transcript transcriptional start sites (TSS) of either protein coding genes or lncRNAs. As expected, for upregulated protein coding genes we observed a general increase of downstream sequencing reads on the sense strand but little indication of significant activity or changes in transcriptional levels either upstream from the TSS or on the anti-sense strand (Fig 1B; left panel). In contrast, for upregulated lncRNAs, in addition to the expected increase in downstream sense strand transcription, we also observe a high level of sequencing reads upstream from the TSS on the sense strand (Fig 1B, right panel). This indicates the presence of upstream sense strand transcriptional activity in a large proportion of the lncRNAs. To further investigate this phenomenon we plotted the average read densities around the TSS of individual genes and subjected these to cluster analysis (Fig 1C). There was generally little evidence of transcriptional activity on the sense strand upstream from the TSS of protein coding genes. However, a large number of lncRNA transcripts showed evidence for upstream transcriptional activity on the sense strand of the lncRNA encoding genes (Fig 1C, bottom panel). This indicates the presence of upstream transcriptional units originating from coding genes as exemplified by the *DUSP5* locus (Fig 1D, top panel). Here, both the *DUSP5* locus and the downstream lncRNA are EGF-inducible. Little anti-sense activity was detected around the transcriptional start sites for both protein coding and lncRNA genes (Fig 1C, right panels). However, there are examples of lncRNAs that are closely positioned in the anti-sense direction downstream from protein coding genes as observed at the *EDN1* locus. This gene is EGF-inducible, and at this locus, the downstream anti-sense lncRNA transcript (termed *EINCR2*) is also EGF inducible (Fig 1D, bottom panel).

We also examined the RNA levels surrounding genes which are downregulated following EGF treatment. Very few protein coding genes are downregulated but a large number of lncRNAs are downregulated (see S1B Fig). In contrast to the EGF-induced lncRNAs, there is evidence for large amounts of anti-sense transcription in the region upstream from the TSS of many EGF-downregulated lncRNAs, suggesting the existence of divergent promoters (S1A and S1B Fig). Collectively, this data indicates that many EGF-regulated lncRNAs form a subclass that is located immediately up or downstream from other transcriptionally active genes. In the case of the EGF-inducible lncRNAs, the existence of upstream sense strand transcription units is suggestive of polymerase run-ons from the upstream gene, a phenomenon that has recently been reported by others [15].

We focussed on the EGF upregulated transcripts and subclassified the lncRNAs according to known genomic annotations or by belonging to the “polymerase run-on” or “anti-sense” classes identified above (Fig 1E).173 of these transcripts (55%) are non-coding RNAs, and of these 67 (21%) are known pseudogenes or are derived from “polymerase run-ons”. The rest of the lncRNAs can be subdivided into either antisense or intronic lncRNAs or long intergenic non-coding RNAs (lincRNA). We identified 13 known lincRNAs and 76 novel lincRNAs. To validate these findings, we performed RT-qPCR on a selection of EGF up- and down-regulated lincRNAs and observed the expected patterns of regulation (Fig 1F). The kinetics of activation and repression varied across a 6 hour time course depending on the lincRNA studied. For example, XLOC-036745 is rapidly induced and rapidly returns to basal levels within 1 hour of stimulation whereas XLOC-014669 (*EINCR1*) shows more sustained activation beyond 90 mins.

We have therefore identified a large number of lncRNAs that are either up or downregulated following EGF stimulation that can be subcategorised into different classes. Due to its rapid and sustained induction kinetics and also its genomic location which is clearly distinct from any neighbouring genes, we decided to focus on characterising one of the lincRNAs *EINCR1*.

### *EINCR1* is an EGF regulated lincRNA

**Fig 2:**
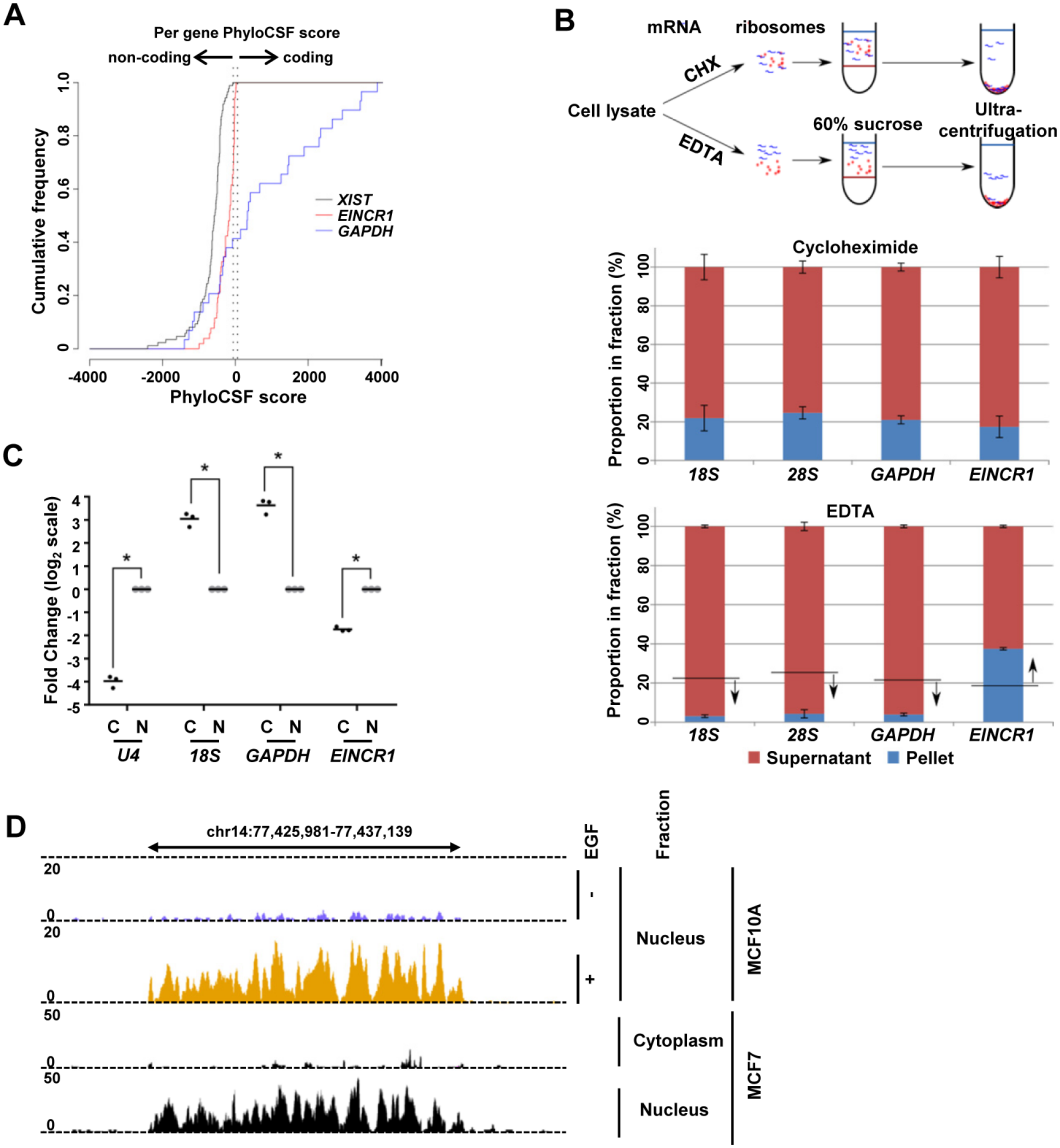
*EINCR1* is an EGF-regulated, nuclear long intergenic non-coding RNA. (A) Cumulative phyloCSF scores of *EINCR1*, *GAPDH* and *XIST*. (B) Top: Schematic of sucrose differential centrifugation of MCF7 cells in the presence of the protein synthesis inhibitor (cycloheximide; ‘freezes’ RNA on the ribosomes) or in the presence of 50 mM EDTA (dissociates ribosomal subunits). Ribosomal subunits are shown in red and RNA species in blue. Bottom: RT-qPCR analysis of relative RNA levels (after 60 min EGF stimulation) in the ribosomal (pellet; blue) and soluble (supernatant; red) fractions in the presence of cycloheximide (top) or EDTA (bottom). Data represent average ±SEM of the proportion in fractions from two biological repeats. (C) RT-qPCR of nuclear (N) and cytoplasmic (C) fractions of MCF10A cells. MCF10A cells were stimulated with EGF for 30 minutes before harvesting RNA. Individual data points from 3 independent repeats were normalized to the nuclear RNA level (taken as 1) and are shown on log_2_ scale. Horizontal lines indicated mean value. * = P-value < 0.01 (t-test with multiple testing correction). (D) UCSC genome browser tracks of *EINCR1* expression in MCF10A (top) in the presence and absence of EGF stimulation (nuclear RNA-seq, this study) and MCF7 (bottom) cells (cytoplasmic and nuclear fractions, ENCODE [1]).

*EINCR1* is located >50 kb from the nearest annotated coding gene, indicating that it should be classified as a long intergenic non-coding RNA (lincRNA). To establish whether *EINCR1* is a true non-coding RNA, we examined its coding potential using PhyloCSF [16]. *EINCR1* scored as a non-coding RNA like *XIST* rather than a protein coding gene such as *GAPDH* (Fig 2A). Next we asked whether *EINCR1* is associated with the ribosomal fraction, and hence its likelihood for undergoing translation. In the presence of cycloheximide, there was little difference between *EINCR1* levels and *GAPDH* levels associated with the ribosomal fraction (Fig 2B, top). However, upon treatment with EDTA, *GAPDH* was released from the ribosome as expected for an RNA undergoing translation upon ribosomal subunit dissociation, whereas the relative levels of *EINCR1* increased, suggesting that any association was likely due to contamination during the fractionation process, rather than undergoing active translation. These results therefore indicate that EINCR1 is a non-coding RNA but we cannot rule out that under some circumstances, short peptides might be generated from the EINCR1 transcript.

Having established that *EINCR1* is likely a non-coding RNA, we next investigated its subcellular localisation. We isolated RNA from the nuclear and cytoplasmic compartments and verified their validity by demonstrating an enrichment of U4 snRNA in the nucleus and 18S rRNA in the cytoplasm (Fig 2C). As expected, the mRNA from protein coding gene is enriched in the cytoplasm. However, *EINCR1* exhibits reciprocal enrichment and was found predominantly in the nuclear fraction (Fig 2C). This nuclear localisation was verified by analysing the expression of *EINCR1* in published RNAseq data from the MCF7 breast cancer cell line (Fig 2D) and is consistent with a previous study that also showed that this lincRNA is localised to the nucleus in PTEC kidney cells [17]. Thus, in common with many lincRNAs, *EINCR1* exhibits a predominantly nuclear localisation.

### *EINCR1* transcription is regulated via the ERK MAPK pathway

**Fig 3:**
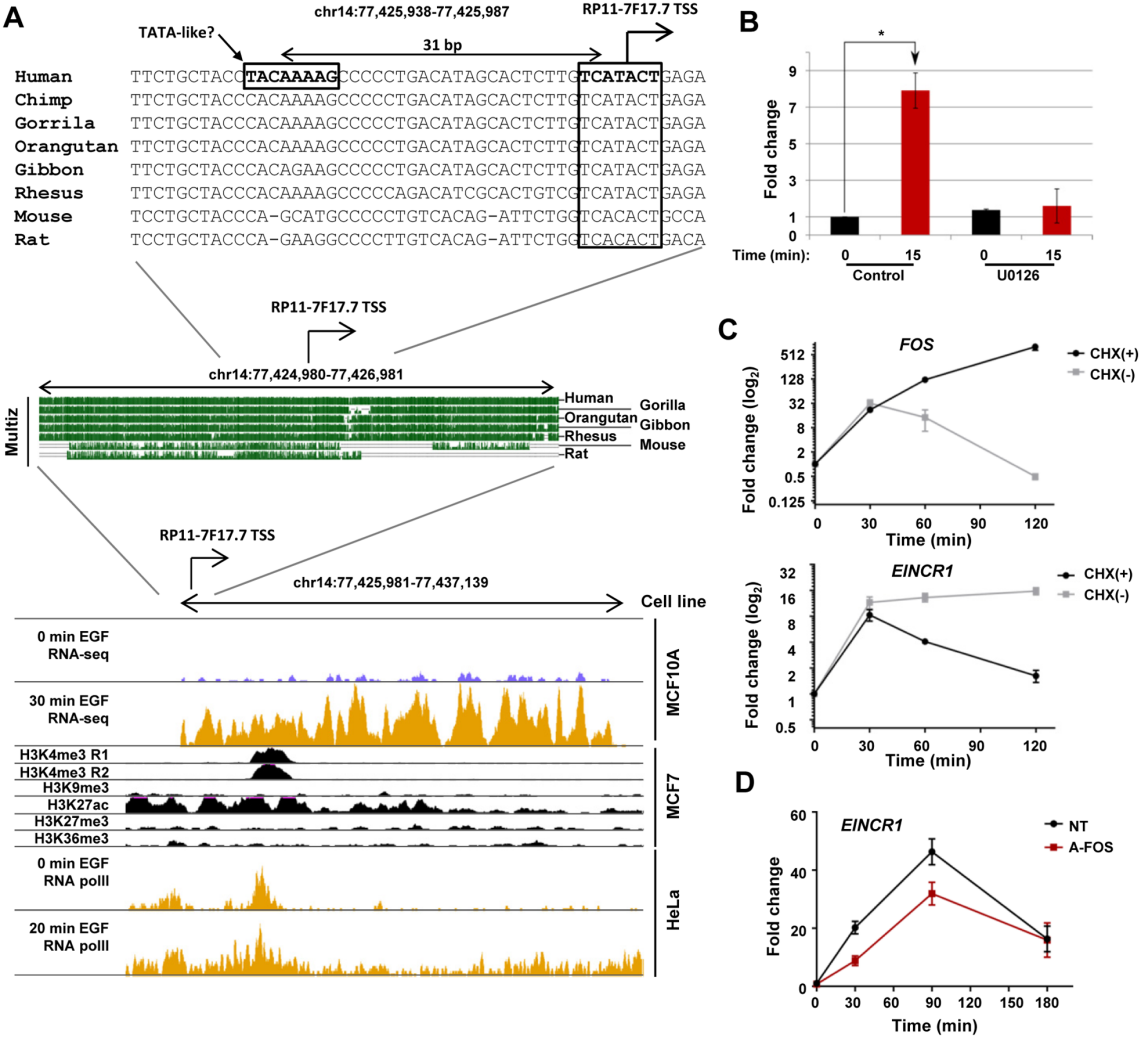
EINCR1 transcription is regulated via the ERK MAPK pathway. (A) A screenshot of the genomic features observed near the *EINCR1* genomic locus compiled from UCSC genome browser data. Data for the histone modifications (H3K3me3, H3K9me3, H3K27ac, H3K27me3, H3K36me3 ChIP-seq are derived from ENCODE data from the MCF7 cell line [38]). RNA polII ChIP-seq data from HeLa cells (before and after EGF induction) are derived from [39]. The RNA-seq data are from this study. The promoter sequence of human RP11-7F17.7 (TSS±1000bp) is aligned with five primates, mouse and rat reference sequence using multiz aligner [40] (middle panel). The sequence immediately surrounding the TSS is shown at in the top panel and a putative TATA-like sequence unique to humans is indicated. The TSS is indicated by an arrow. Genomic locations are shown above each panel. (B) RT-qPCR analysis of *EINCR1* expression following EGF treatment of MCF10A cells for 15 mins in the presence and absence of the MEK inhibitor U0126. n = 2, * = P-value < 0.05. (C) RT-qPCR analysis of *FOS* and *EINCR1* after EGF stimulation of MCF10A cells for indicated times in the presence or absence of cycloheximide (CHX). Data are shown relative to the zero timepoint (taken as 1) and represent mean ± SEM from two independent repeats. (D) RT-qPCR analysis of *EINCR1* after EGF stimulation of MCF10A cells for the indicated times in the presence or absence of dominant negative FOS (A-FOS). Data are shown relative to the zero timepoint (taken as 1) and represent mean ± SEM from two independent repeats.

To examine *EINCR1* expression in more detail we initially delineated the transcript structure. First we compared the extent of the transcription unit we had identified with the annotated transcripts in this genomic region. Two putative non-coding RNAs, RP11-7F17.7 and RP11-7F17.1 (also known as LINC01629 or linc-KIAA1737-2) had previously been documented in this region (S2A Fig). Our RNAseq data provide supporting data for the existence of two of the annotated exons, indicating that transcript splicing occurs and the *EINCR1* transcription unit appears to span these two loci (S2A Fig). We confirmed the existence of the splice junction by RT-PCR and Sanger sequencing. Next we established the transcriptional start site (TSS) of the *EINCR1* lincRNA by 5’ RACE. The start site we identified is located two bp upstream from the one identified for RP11-7F17.7 and corresponds exactly with the most common start site identified in CAGE data from cell lines examined by the FANTOM5 consortium (S2B Fig; [18]). The region surrounding this TSS is highly conserved amongst other primates and is also well conserved with rodents (Fig 3A). However, while the promoter region is conserved, the region further downstream from the TSS shows much lower conservation in rodents, suggesting a lack of functional conservation for the lincRNA (Fig 3A, middle). There is a putative TATA-like element which is specific to humans and is potentially associated with promoter functionality in this species. We also examined the histone marks which are associated with the locus in MCF7 cells and found evidence for H3K4me3 and H3K27ac which demarcate active chromatin regions but little sign of the repressive marks H3K9me3 or H3K27me3 (Fig 3A, bottom). However, the H3K4me3 peak is not located close to the TSS as commonly found in active genes and the H3K27ac mark is spread over a wide area rather than delineating a single promoter or enhancer unit. We also examined RNA polymerase II ChIP-seq data from EGF stimulated HeLa cells and found regions of enrichment around the TSS and H3K4me3 peak which increased in magnitude following EGF induction and spread across the entire locus (Fig 3A, bottom), consistent with the EGF-inducible transcription of *EINCR1* that we observe in MCF10A cells. Finally we examined *EINCR1* expression in two breast cancer cell lines and showed that, similar to *FOS*, it is inducible by EGF stimulation in MCF7 cells but not in MDA-MB-231 cells (S2C Fig).

Next we asked whether EGF-mediated *EINCR1* expression is mediated through the ERK MAP kinase signalling pathway. MCF10A cells were stimulated with EGF in the presence or absence of the MEK inhibitor U0126. EINCR1 was efficiently induced following EGF treatment for 15 mins but this response was abolished upon MEK inhibition (Fig 3B), indicating that *EINCR1* is induced through ERK pathway signalling. Other EGF-inducible genes like *FOS* belong to the immediate-early class of genes and can be activated in the absence of new protein synthesis [19, 20]. We therefore tested whether *EINCR1* expression is dependent on new protein synthesis by treating cells with cycloheximide prior to EGF stimulation. As expected, initial *FOS* induction proceeded as normal but expression was prolonged in the presence of cycloheximide due to the lack of feedback inhibition on its expression (Fig 3C, top). In contrast, although initially *EINCR1* induction kinetics were unaffected, sustained *EINCR1* message production is lost in the presence of cycloheximide, indicating that new protein synthesis is essential for the second phase of *EINCR1* induction (Fig 3C, bottom). EGF stimulation leads to the production of new AP1 complex components such as FOS (reviewed in [13]) which might contribute to this later phase of *EINCR1* induction. Indeed there are two evolutionarily conserved AP1 binding sites within the 250 bp region upstream from its TSS (S2D Fig). To establish whether AP1 might be playing a role in controlling *EINCR1* expression, we transduced MCF10A cells with a lentivirus containing a dominant-negative version of FOS (A-FOS; [21]). Compared to control treated cells, the levels of *EINCR1* were reduced following EGF treatment in the presence of A-FOS (Fig 3D).

Collectively, these data demonstrate that *EINCR1* expression is activated by EGF through the ERK pathway and that the transcription factor AP1 plays a role in its activation.

### *EINCR1* expression patterns in tissues and cancer

**Fig 4:**
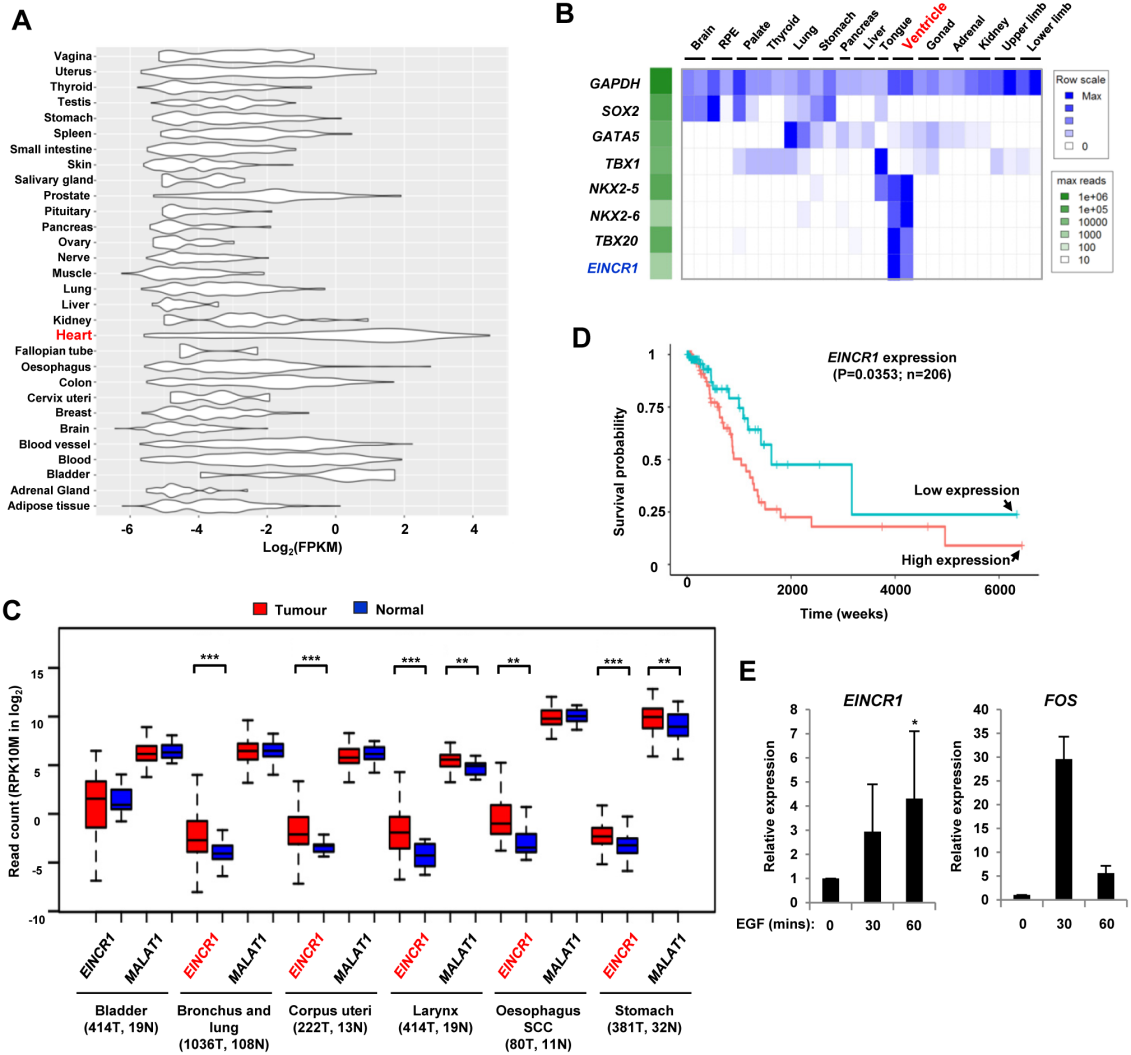
*EINCR1* is specifically expressed in normal human heart tissues and is up-regulated in cancer. (A) Violin plots of the expression of RP11-7F17.7 in normal tissues [37]. (B) Heatmap showing the expression of *EINCR1* and the indicated protein coding genes across different organs in human embryos [22]. Data are row-normalised to the maximum observed expression (blue scale). The maximum absolute expression of each gene is indicated by the green scale. (C) Boxplots of *EINCR1* and *MALAT1* expression in the indicated cancer categories. Expression in both normal (N; blue) and tumour (T; red) samples are shown. Numbers of each type of sample are provided below each cancer subtype. Median values are shown by horizontal lines. Statistically significant differences are indicated: ** = P-value <0.01; *** = P-value<0.001. (D) Kaplan-Meier plot of overall survival in patients with lung adenocarcinoma (n = 206, from the TCGA-LUAD dataset)), using sample groups with either the top or bottom 20% expression of *EINCR1*. Log rank probabilities between low and high expression are shown. (E) RT-qPCR analysis of *EINCR1* and *FOS* expression after EGF stimulation of A549 cells for the indicated times. Data are shown relative to the zero timepoint (taken as 1) and represent mean ± SD (n = 3). * = P-value = 0.05.

To begin to understand the biological function of *EINCR1*, we first established its expression pattern in human tissues. In the adult, *EINCR1* was generally expressed at a low level across the majority of tissues. However, high level expression is observed in the heart and to a moderate level in the bladder (Fig 4A). We also examined *EINCR1* expression in the developing human embryo [22] and its expression is exclusively detected in the heart ventricle, alongside other heart-specific genes such as *NKX2*.*5*, *NKX2*.*6* and *TBX20* (Fig 4B). Therefore, under normal physiological conditions, *EINCR1* likely functions in the context of the heart.

To establish a potential disease link, we next looked for aberrant *EINCR1* expression in cancer and interrogated 10,406 samples generated by the TCGA Research Network [23]. Little difference was seen for many cancers as illustrated by bladder cancer (Fig 4C). However, several other cancers showed enhanced expression of *EINCR1* compared to normal tissue, as exemplified by the “bronchus and lung” category (Fig 4C). Expression of the lincRNA *MALAT1* is shown for comparison, and although upregulation is seen in some cancers, there is little difference in the “bronchus and lung” group. Further sub-partitioning of the “bronchus and lung” category showed that *EINCR1* is particularly overexpressed in several cancer subgroups, including squamous cell carcinomas and adenocarcinomas (S3A Fig). We further investigated this phenomenon by studying the expression of *EINCR1* and other EGF-inducible lncRNAs in an independent dataset from lung adenocarcinomas [24]. The majority of EGF inducible protein coding genes show a general reduction in expression in these cancer samples (as exemplified by *FOS*) (S3B Fig), and the same was observed for EGF-inducible lncRNAs (S3C Fig). However, a subset of lncRNAs, including *EINCR1*, shows upregulation in lung adenocarcinomas. Given this strong association of *EINCR1* expression with lung cancer, we investigated whether *EINCR1* expression correlated with disease prognosis in lung adenocarcinoma [25]. High level *EINCR1* expression gave a significantly worse disease prognosis with patients exhibiting high level expression showing lower long term survival (Fig 4D). As we initially identified *EINCR1* in breast epithelial cells, we tested whether we could observe inducible expression in A549 lung adenocarcinoma cells. EGF treatment caused the expected transient induction of *FOS* in these cells and also a more sustained activation of *EINCR1* (Fig 4E) in these lung adenocarcinoma cells.

Together these results demonstrate that during normal development, *EINCR1* is expressed in a tissue-specific manner and is largely confined to the heart. However, *EINCR1* is aberrantly expressed in several cancers, and in the case of lung adenocarcinoma, high level expression correlates with poor prognosis.

### High level *EINCR1* expression reduces EGF-regulated protein coding gene expression

**Fig 5:**
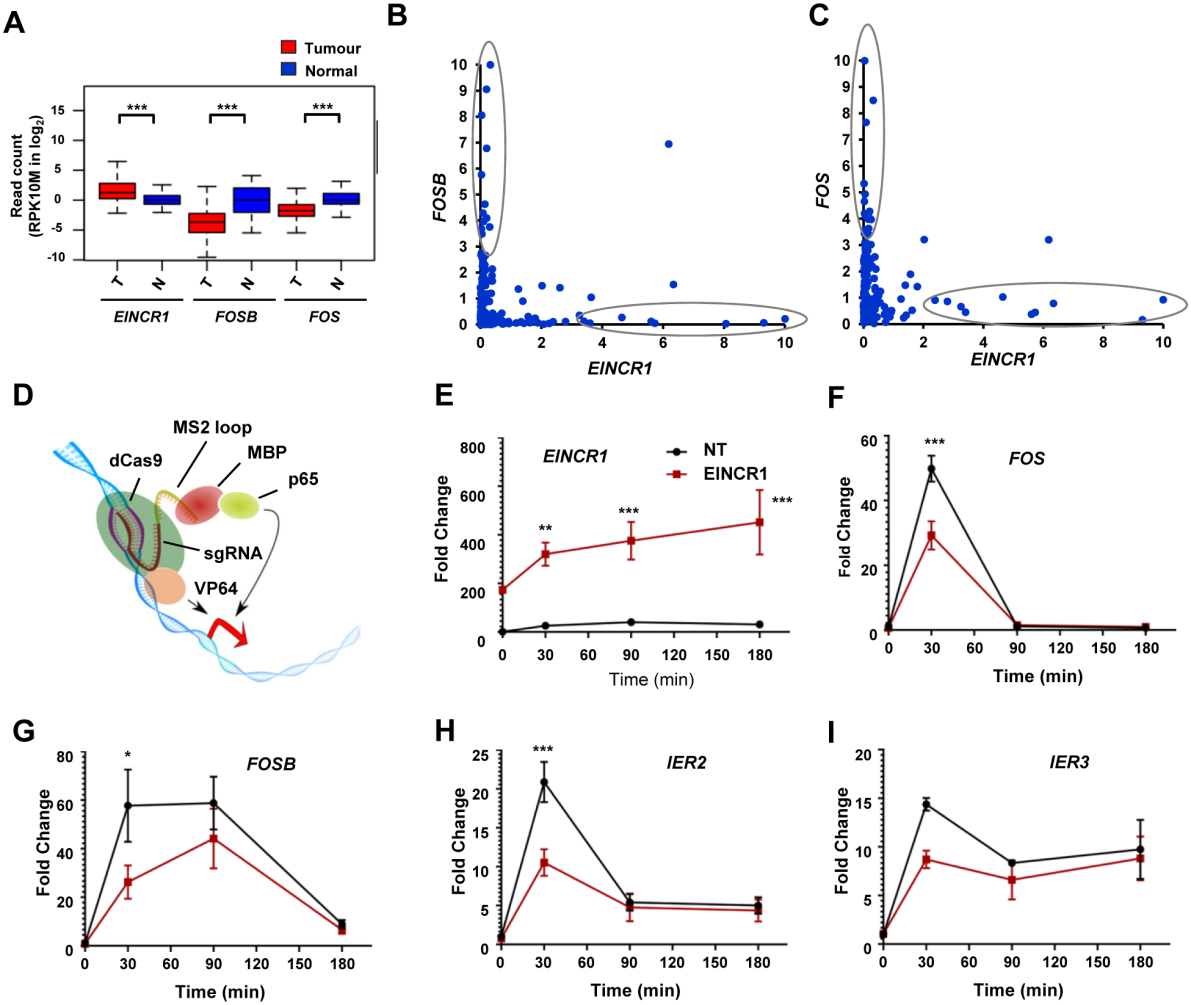
High levels of *EINCR1* transcription leads to changes in the expression profiles of EGF-regulated genes. (A) Boxplots of *EINCR1*, *FOSB* and *FOS* expression in lung adenocarcinomas (n = 339 cancer samples from lung adenocarcinomas “not otherwise specified” [NOS], and 108 normal lung samples from TCGA data). Expression in both normal (N; blue) and tumour (T; red) samples are shown. Median values are shown by horizontal lines. Statistically significant differences are indicated: *** = P-value<0.001. (B and C) Scatterplots showing the expression of *EINCR1* and either *FOSB* (B) or *FOS* (C) in lung adenocarcinomas NOS (n = 339). Samples containing either high *EINCR1* but low protein coding gene expression (or vice versa) are circled. The units on each axis are the RPK10M (reads per 1 kb per 10 M reads) normalised by the maximum value of the corresponding gene and then multiplied by 10. (D) Model of the CRISPR-based upregulation system [26]. dCas9 –mutated Cas9, VP64—4x viral protein 16 transactivation domain, MBP—MS2 binding protein, p65 –transcriptional activation domain of p65. (E-I) RT-qPCR analysis of expression of *EINCR1* (E) or the indicated protein coding genes (F-I) following EGF stimulation of MCF10A cells for the indicated times either in the presence of guide RNA targeting the *EINCR1* promoter 218 bp upstream of the TSS (red line) or control non-targeting (NT) guides (black line). Data are shown relative to each sample in the absence of EGF (taken as 1) and are from three independent replicates (except *IER3* where n = 2). * = P-value <0.05; ** = P-value <0.01; *** = P-value<0.001.

We noticed that in lung adenocarcinoma samples, many EGF-inducible genes, including *FOS* and *IER2* are downregulated whereas *EINCR1* expression is generally upregulated (S3B and S3C Fig). This suggests a potentially reciprocal relationship between *EINCR1* and other EGF-regulated genes. To further explore this phenomenon, we analysed the expression of *EINCR1* and two EGF-inducible genes, *FOS* and *FOSB* across the lung adenocarcinoma samples in the TCGA-LUAD dataset. While *EINCR1* is upregulated in the cancer samples, both *FOS* and *FOSB* show reduced expression in cancer samples (Fig 5A). When analysed on a sample by sample basis, we observed that when *EINCR1* expression is high, then the expression of the coding gene is almost always low and vice versa (Fig 5B and 5C). There is therefore a reciprocal relationship between *EINCR1* and the expression of EGF regulated protein coding genes and suggests that the high levels of *EINCR1* might be responsible for dampening down their expression. To test this we used a CRISPR-derived system (Fig 5D; [26]) to drive constitutive overexpression of *EINCR1* in MCF10A cells. Efficient *EINCR1* overexpression was achieved (Fig 5E) and in the presence of enhanced *EINCR1* levels, the expression of the EGF regulated genes, *FOS*, *FOSB*, *IER2* and *IER3* was dampened (Fig 5F–5I).

Collectively these data demonstrate overexpression of *EINCR1* leads to defects in the induction of EGF-activated protein coding genes and likely explains the reciprocal relationship between *EINCR1* expression and the expression of genes like *FOS* and *FOSB* in cancer cells.

## Discussion

It is now appreciated that lncRNAs are important regulatory molecules that play a role in controlling a variety of cellular activities [3]–[6]. Here we have identified a large number of lncRNAs whose expression is altered in response to signalling pathway activation following EGF treatment. These lncRNAs belong to different categories but the major class is lincRNAs, of which the majority are novel transcripts. One interesting additional class we identified corresponds to the previously identified “polymerase run-ons” which occurs at the 3’ ends of genes where the transcript extends beyond the usual termination site [15]. In the case of *DUSP5*, this could be explained by inefficient transcriptional termination in the face of high levels of EGF-induced transcription. However, there are several instances where the expression of the upstream coding region is unaffected but the “run-on” transcription is enhanced following EGF induction. It is not clear what mechanism is involved in lncRNA induction at these loci, nor is it clear what the functional significance of these events is.

We focussed on one of the EGF inducible lincRNAs, *EINCR1*. This lincRNA is rapidly induced following EGF induction but unlike typical EGF-inducible protein coding genes such as *FOS*, it exhibits extended activation kinetics. *FOS* is classified as an immediate-early gene due to the lack of requirement for protein synthesis for its induction and subsequent super induction following treatment with the protein synthesis inhibitor cycloheximide. However, while *EINCR1* is still induced in the presence of cycloheximide, its prolonged activation is lost, indicating that it is not susceptible to the same negative feedback loops as *FOS* and requires new protein synthesis for its sustained activation. One such factor appears to be AP1 family transcription factors, several of which are inducible by EGF (including FOS, FOSB, JUN and JUNB). This therefore suggests an interesting regulatory network in which EGF-inducible protein coding genes like *FOS* cause increases in intracellular AP1 levels that enhance the expression of *EINCR1* which in turn could act to dampen down any further activation of EGF-coding genes.

Our data indicate that *EINCR1* is predominantly nuclear although ideally future studies should utilise additional approaches such as FISH to confirm this and detect potential subnuclear localisation. However its nuclear localisation suggests that *EINCR1* might have a role in controlling gene expression either directly or indirectly through alterations to the underlying chromatin state. Our initial studies point to such a possibility as overexpression of *EINCR1* leads to decreased expression of a cohort of EGF-induced protein coding genes such as *FOS* and *FOSB*. This observation is consistent with the observation that in several human cancer subtypes, *EINCR1* is overexpressed, and in the case of lung adenocarcinomas its expression is anti-correlated with the expression of these genes. Thus, one function of *EINCR1* might be to dampen down the levels of gene induction in response to EGF pathway activation. In humans, *EINCR1* is predominantly expressed in the heart, and future studies are needed to study whether *EINCR1* plays a role in modulating the output from receptor tyrosine kinase pathway signalling in this context. Although not highly expressed in the majority of tissues, our observation that *EINCR1* expression is EGF inducible might reflect low basal level expression and yet the potential for activation under defined signalling conditions in a wider cellular context. Moreover, induction by other signalling events might also broaden its expression repertoire and a lincRNA transcribed from the same locus (KIAA1737-2), has been shown to be induced by cytokine treatment of human PTEC kidney cells [17]. EINCR1/ KIAA1737-2 might therefore by a commonly upregulated gene that responds to many different stimuli.

*EINCR1* is upregulated in several different cancer subtypes, including lung adenocarcinomas. This is not a general phenomenon shared by all lincRNAs as *MALAT1* is often associated with cancer progression (reviewed in [27]) but is only upregulated in a subset of the cancers where *EINCR1* is overexpressed. It is not clear what role *EINCR1* might have in the tumourigenic process but as *EINCR1* is a target of the EGF pathway and EGFR-regulated pathways are often activated in cancer cells it appears likely that it might be an important mediator of dysregulated signalling. The reciprocal expression of *EINCR1* and other EGF-regulated protein coding genes suggests a functional relationship which is reinforced by our over-expression studies. Additional work will be needed though to investigate the relevance of this relationship in the context of cancer.

In summary, we have identified a large number of EGF inducible lincRNAs and shown that one of these, *EINCR1*, is potentially important in the context of human cancer. Our findings provide a useful resource for further investigation of both *EINCR1* and the dozens of other lncRNAs we have identified in modulating the output of EGF pathway signalling.

## Materials and methods

### Cell culture

MCF10A cells were grown in DMEM/F12 (Gibco, 11320–033) containing 5% horse serum (Biosera, DH291), 20 ng/ml EGF (Sigma, E1257), 10 μg/ml insulin (Sigma, I0516), 100 ng/ml cholera toxin (Sigma, C9903) and 0.5 μg/ml hydrocortisone (Sigma, H0396) (complete medium).

A549, MCF7, MDA-MD-231 and HEK293T cells were grown in either RPMI 1640 with L-glutamine, 25mM HEPES (for A549 cells)(Life Technologies, 52400041) or DMEM (Gibco, 22320–22) supplemented with 10% fetal bovine serum (Life Technologies, 10270–098). All of the cells were grown up to 90% confluence and were passaged every 2–3 days by brief washing of the cells with Dulbecco's phosphate-buffered saline (DPBS, Life Technologies, 14190–094), detached with Trypsin-EDTA (0.05%) (Life Technologies 25300–054) and replated at 1 in 5 dilution in full media. Where indicated, the MEK inhibitor U0126 was used at a final concentration of 10 μM in 0.1% DMSO and the protein synthesis inhibitor cycloheximide at 25 μg/ml. All cell lines were obtained from ATCC except A549 cells which were a gift from Caroline Ridley. We verified the identity of the genotypes of all the cell lines we used.

### Nuclear RNA-seq

1.5 x 10^6^ MCF10A cells were seeded onto a 60 mm dish in complete media without EGF and with 0.5% horse serum instead of 5% (see section 2.1). After 48 hrs incubation, EGF was added to a final concentration of 20 ng/ml. After 30 minutes, nuclear fractions were isolated according to the protocol described previously [1]. At the same time, nuclear fractions were isolated from cells which were not stimulated with EGF. Briefly, cells were washed with 1xPBS and then incubated for 10 min with 1 ml of RLN buffer (50 mM TrisHCl pH = 8.0, 140 mM NaCl, 1.5 mM MgCl_2_, 0.5% (v/v) IGEPAL CA-630 and 2 U/ml SUPERaseIn—RNase inhibitor, Invitrogen AM2696). Subsequently, the lysate was centrifuged at 1,000 g for 5 min at 4°C, the pellet was collected, resuspended in 1 ml of RLN buffer, incubated for a further 5 min on ice and centrifuged at 1 000 g for 5 min at 4°C. At this stage nuclear fractions from different experimental days were stored at -80°C for subsequent RNA extraction. The RNA was extracted from the nuclear fraction using an RNeasy plus kit (Qiagen, 74134) with DNase treatment according to the manufacturer’s protocol. After isolation, the purity and integrity of RNA were investigated using a Bioanalyser. Only non-degraded samples with a ratio of 260/280 nm absorption above 2 were used for sequencing library preparation.

The cDNA libraries were prepared TruSeq Stranded mRNA Sample Prep Kit (Illumina, RS-122-2101) for polyadenylated-tailed RNA sequencing. The preparations of libraries together with subsequent sequencing reactions were performed at the Genomic Technologies Facility, University of Manchester according to the manufacturer’s recommendations. The sequencing reaction was performed on the Illumina HiSeq 2500. Data are deposited in ArrayExpress (E-MTAB-5370).

### RNA extraction and RT-qPCR

RNA was extracted using the RNeasy plus kit (Qiagen, 74134) following the manufacturer’s protocol. Subsequently, RNA samples were quantified using a Nanodrop 2000 (Thermo Scientific) and concentrations were normalized to 20 ng/μl. 40 ng of each sample was used per reverse transcription-quantitative polymerase chain reaction (RT-qPCR) reaction using the QuantiTect SYBR^®^ Green RT-PCR Kit (Qiagen, 204243) on Rotor-Gene Q (Qiagen) real-time PCR machine.

When indicated, the nanolitre volume RT-qPCR was performed using the Fluidigm Biomark HD system using EvaGreen chemistry. The reactions were performed by Claire Morrisroe at The Genomic Facility, University of Manchester following manufacturer’s protocol. The output data were processed following the default quality protocol. Data points with more than one peak in the melt analysis were discarded.

The final results are normalised to the house keeping genes using the delta delta Ct method [28]. S2 Table contains the list of primers used.

### Ribosome analysis—Sucrose cushion

MCF7 cells were plated onto 150 mm petri dishes at the density required for ~70% confluency on the assay day (around 5 x 10^6^ cells). On the day of experiment, cells were stimulated with EGF for the indicated times. Cells were incubated for 5 minutes before lysate collection with 100 μg/ml of cycloheximide (CHX, Sigma, C7698, protein synthesis inhibitor that ‘freezes’ the actively translated mRNA with ribosomes and nascent protein). Subsequently, dishes were transferred onto ice/water bath, media was removed and cells were washed with 1x PBS containing 100 μg/ml of CHX. Cells were gently scraped into 1 ml of 1x PBS and centrifuged at 200 x g for 5 minute at 4°C. Subsequently, cells were resuspended in 200 μl of CSB buffer (300 mM sorbitol, 20 mM HEPES, pH 7.5, 1 mM EGTA, 5 mM MgCl_2_, 10 mM KCl, 10% Glycerol, 100 μg/ml CHX and protease inhibitor cocktail) and the cell membrane was disrupted with glass beads for 45 s. The lysate was cleared by centrifugation at 9,300 g for 10 min. The Optical density (OD) at 260 nm was measured and lysates were normalized for OD. For EDTA treatment, EDTA was added to a final concentration of 50 mM and lysates were overlaid over 60% sucrose solution in CSB buffer (without Sorbitol and protease inhibitors). Samples were centrifuged for 150 min at 4°C at 55,000 g. RNA was collected from the pellet and supernatant fractions (supplemented with luciferase RNA (Promega, L4561)–normalization spike-in) using trizol LS reagent (Life technologies, 10296). RNA levels were quantified with RT-qPCR and normalized to the input.

### Lentiviral transductions

Second generation lentiviral particles were produced as described previously [29] using psPAX2 and pMD2.G packaging plasmids (Addgene 12260 and 12259). The viral particles were concentrated using PEG-it solution (System Biosciences, LV825A-1) and quantified using a qPCR method [30].

Transductions of the human cell lines with concentrated viral particles were performed in 6-well plates. Firstly, cells were plated at ~40% confluency in the media appropriate for the cell line. After 8 hrs, growth media was changed to the fresh growth media supplemented with 10 μg/ml of polybrene (Millipore, TR-1003-G) and the appropriate amount of the viral particles. The following day, the media was changed to the assay media required for the subsequent analysis.

### CRISPR methods

Guide target sequences were designed following the protocol described previously [31]. For the overexpression studies, we used the dCas9-VP64 and MS2-p65 system [26]. Briefly, DNA duplexes corresponding to the guide RNAs were cloned into the BsmBI site of the lenti sgRNA(MS2)_zeo plasmid (Addgene 61427), to give the following plasmid; pAS4516 (containing guide sequence 5-CACCGTTTATCCCAGCATGAGGCG-3’ targeting region 218 nucleotides upstream of the *EINCR1* TSS). Guide RNAs were designed using E-CRISPR (e-crispr.org; [31]) using the strict setting. sgRNA(MS2)_zeo plasmid encoding scrambled guide was use as a control (pAS4519, 5’-CACCGTGGTTTACATGTCGACTAA-3’). iral particles were produced for the sgRNA(MS2) plasmids and for lenti MS2-P65-HSF1_Hygro (Addgene 61426) and lenti dCAS-VP64_Blast (Addgene 61425) and the target cell lines were sequentially transduced with these plasmids. Gene expression analysis was performed using RT-qPCR.

### Computational data analysis

For the nuclear RNA-seq data, raw sequencing files (fastq format) were investigated for the quality of reads, duplication level and GC content using *fastqc* packages [32]. Subsequently, raw reads were processed with the Trimmomatic tool which removed low quality reads and contamination with sequencing adapters [33]. The trimmed sequencing reads were aligned to the Ensembl transcription (release 72, 2013-03-06) and human genome version 19 (hg19) using *RNA-Star* aligner (version 2.3.0e) [34]. The aligner was run with default settings with option:—outFilterMultimapNmax 100, which allows for up to 100 genomic locations per read. Subsequently, reads that were mapped to multiple loci and redundant reads were removed using Pickard tools (http://broadinstitute.github.io/picard/). *De novo* transcriptome assembly was performed with *Cufflinks* package version 2.2.1 and it was run with default setting. Transcripts were identified using *Cuffmerge* packages [35, 36] with default settings and merged with the GENCODE (v. 19) transcriptome. Differential analysis was performed with *Cuffdiff* package where the transcriptome assembled by the *Cuffmerge* package was used as a reference. Subsequently, data quality was analysed using *CummeRbund* package version 2.0.0 [36]. All of the above calculations were performed using the computational shared facility at the University of Manchester. Subsequent data analysis was performed using custom written Perl scripts and R programming language and data were also processed in the *MS Excel* software. *TiBCO Spotfire* software was used for data visualisation.

For the RNA-seq data downloaded from gastric adenocarcinomas [24], the same protocol was applied. The expression of *EINCR1* in normal human tissues was analysed in data downloaded from http://www.gtexportal.org/ (version 6p; [37]). The annotated lncRNA encompassing part of *EINCR1* (*RP11-7F17*.*7*) was used in the search. To study *EINCR1* expression during human organogenesis, embryonic RNA-seq data [22] were mapped to hg38 using STAR [34] to generate gene level (GENCODE 23) read counts. After quantile normalisation of all genes {R package preprocessCore}, selected genes were row-normalised to the maximum observed expression.

In order to obtain the *EINCR1* expression in the samples in RNA-seq data generated by the TCGA Research Network (http://cancergenome.nih.gov/), we first downloaded the bam files of the RNA-seq data from the data portal of the NCI Genomic Data Commons (GDC) [23]. Then from each bam file we counted the number of reads overlapping with the genomic region occupied by *EINCR1* and used the number of reads per 1kb exonic genomic region per 10M reads (RPK10M) as the *EINCR1* expression in the corresponding sample. The expression of other genes such as *FOS*, *FOSB* and *MALAT1* were also obtained in the same way.

The RNA level in all of the RNA-seq based experiments is presented as fragments per kilobase per million reads (FPKM) or per 10 million reads (FPK10M).

## Supporting information

S1 Fig EGF-stimulation leads to transcriptional downregulation of lncRNAs. (PDF)

S2 Fig Gene structure of *EINCR1*. (PDF)

S3 Fig *EINCR1* is up-regulated in lung cancer samples. (PDF)

S1 Table RNAseq data from EGF stimulated MCF10A cells. (XLSX)

S2 Table List of primers used. (XLSX)
