# Supplementary material for: EINCR1 is an EGF inducible lincRNA overexpressed in lung adenocarcinomas
Source: PLoS One. 2017 Jul 21;12(7):e0181902. doi: 10.1371/journal.pone.0181902 (PMC5521836; doi:10.1371/journal.pone.0181902)
Supplement: S1 Fig — (PDF) [file pone.0181902.s001.pdf]

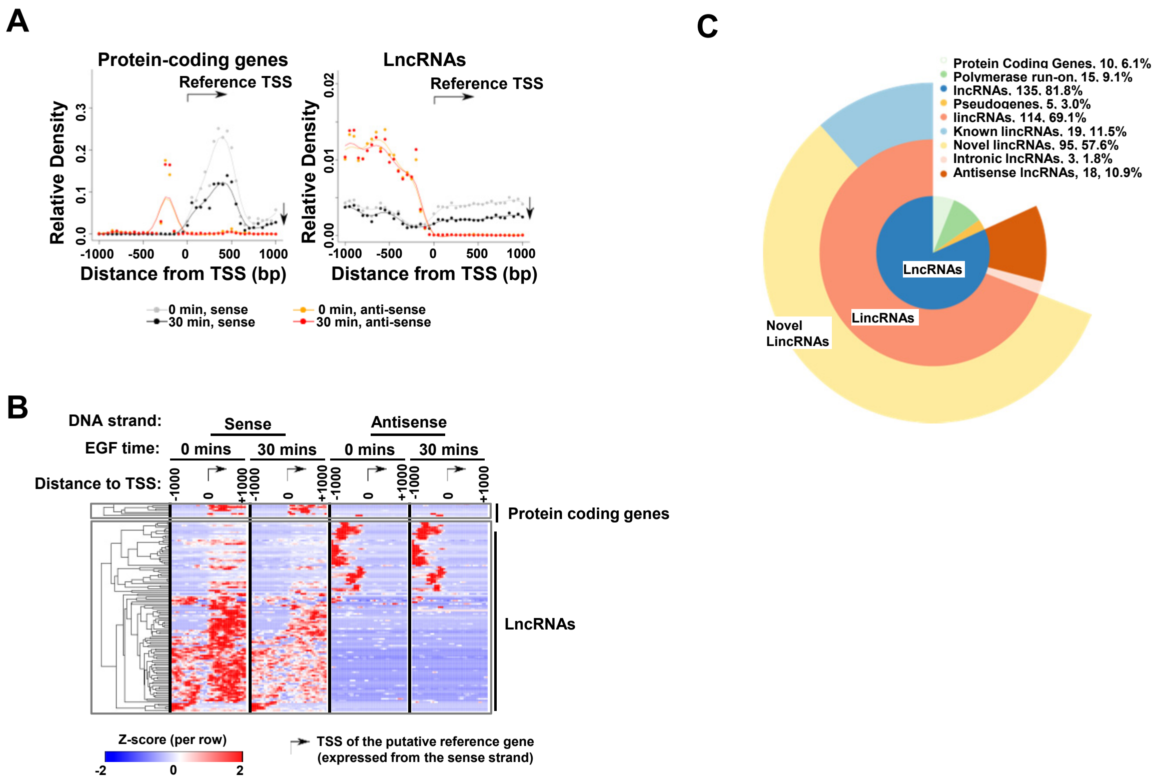

S1 Fig. EGF-stimulation leads to transcriptional downregulation of lncRNAs. (A) Average profiles of sequencing read density in the 2000 bp window around the putative transcription start sites (TSS) of EGF-downregulated protein-coding genes and lincRNAs. (B) Heat map showing the sequencing read density in the 2000 bp window around the putative transcription start sites of EGF-downregulated protein-coding genes and lincRNAs. (C) Distribution of EGF-downregulated genes across gene classes defined by the ENCODE project.
