# Supplementary material for: EINCR1 is an EGF inducible lincRNA overexpressed in lung adenocarcinomas
Source: PLoS One. 2017 Jul 21;12(7):e0181902. doi: 10.1371/journal.pone.0181902 (PMC5521836; doi:10.1371/journal.pone.0181902)
Supplement: S2 Fig — (PDF) [file pone.0181902.s002.pdf]

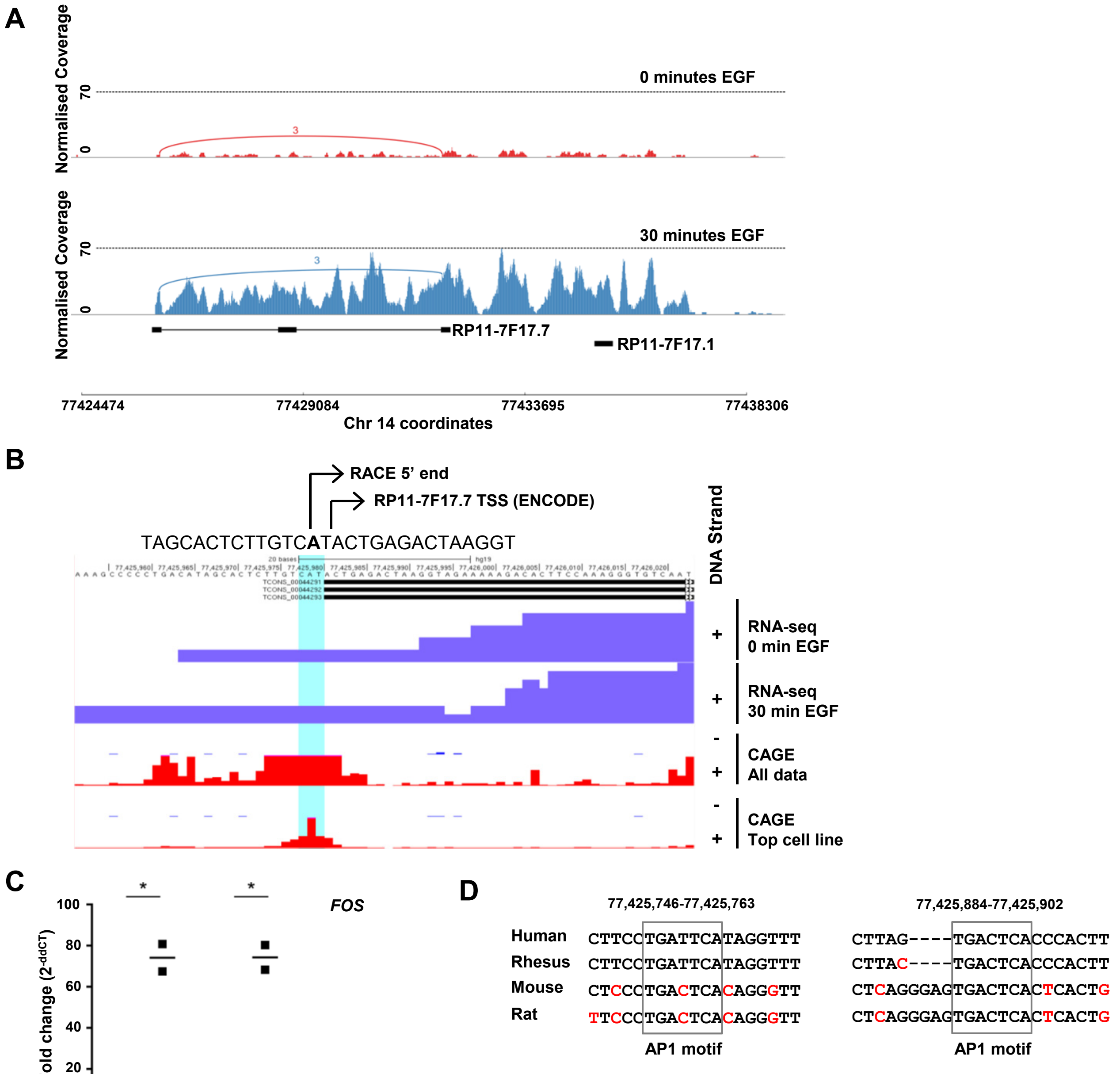

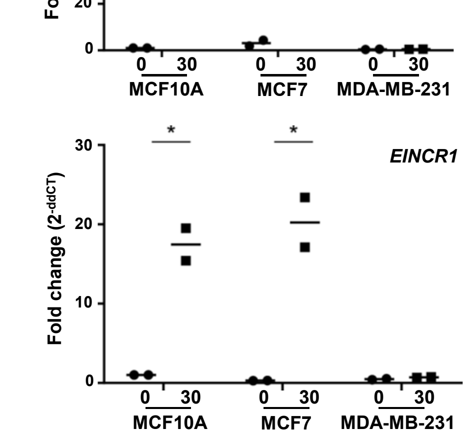

S2 Fig. Gene structure of EINCR1. (A) Sashimi plot of the splice junctions detected in the nuclear RNA-seq data. The coverage represents raw sequencing data for the *EINCR1* genomic locus. Lines indicate detected splice junctions together with the number of reads supporting the junction. Only junctions with at least 2 reads are shown. The gene structures for GENCODE RP11-7F17.7, RP11-7F17.1 and EINCR1 exons identified using RT-PCR are shown together with their genomic locations (hg19 assembly). (B) Location of the 5'-end of EINCR1 based on the 5' RACE. A nucleotide (bold) in the known genomic sequence indicates the TSS of EINCR1 (identified in 5' RACE). The location of the EINCR1 TSS was overlaid with the RNA-seq and CAGE data from FANTOM5 consortium (Forrest et al., 2014). "CAGE all data" – combined all CAGE reads from all studies cell lines. "CAGE top cell line" – data from the cell line with the highest read number in the depicted window. (C) RT-qPCR analysis of *FOS* and *EINCR1* expression in MCF10A, MCF7 and MDA-MB-231 cell lines. Cells were starved of EGF for 48 hrs and subsequently stimulated with EGF for 30 minutes. Individual data points from 2 independent repeats were normalized to the 0 time point of MCF10A cells (taken as 1). (D) Sequence alignments of sequences surrounding AP1 motifs in the promoter region of *EINCR1* in the indicated organisms.
