# Supplementary material for: EINCR1 is an EGF inducible lincRNA overexpressed in lung adenocarcinomas
Source: PLoS One. 2017 Jul 21;12(7):e0181902. doi: 10.1371/journal.pone.0181902 (PMC5521836; doi:10.1371/journal.pone.0181902)
Supplement: S3 Fig — (PDF) [file pone.0181902.s003.pdf]

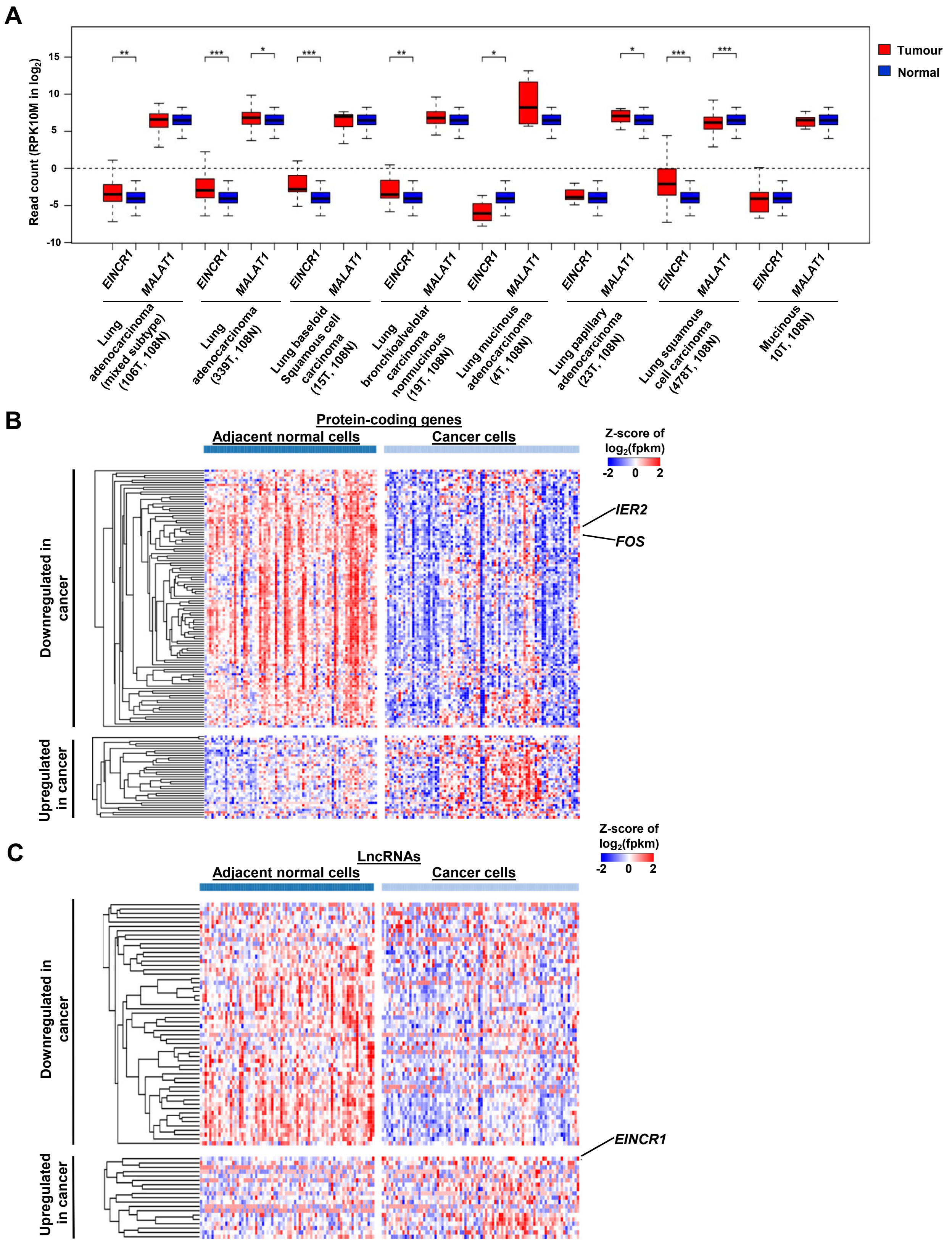

S3 Fig. *EINCR1* is up-regulated in lung cancer samples. (A) Boxplots of *EINCR1* and *MALAT1* expression in the indicated cancer subtypes. Expression in both normal (N; blue) and tumour (T; red) samples are shown. Median values are shown by horizontal lines. Statistically significant differences are indicated: *= P-value <0.05; **= P-value <0.01; ***= P-value<0.001. (B and C) Heatmaps showing the expression of EGF-inducible protein coding genes (B) or lncRNA genes (C) in primary lung cancer samples and adjacent normal cells (Seo et al., 2012). Each row indicates a gene. The expression data are normalised per row as a z-score of the expression. Hierarchical clustering was used to cluster the gene expression profiles. Two major clusters are identified which correspond to genes upregulated or downregulated in cancer. For the lincRNA, only genes with FPKM (in at least one condition) above 0.1 are shown. Rows corresponding to *FOS*, *IER2* and *EINCR1* are indicated.
